# Severe pulmonary arterial hypertension associated with congenital cardiac shunts: evolution under specific treatment

**Published:** 2017

**Authors:** RI Negoi, I Ghiorghiu, F Filipoiu, M Hostiuc, I Negoi, C Ginghina

**Affiliations:** * Anatomy Department, “Carol Davila” University of Medicine and Pharmacy, Bucharest, Romania; ** Cardiology Department, “C.C. Iliescu” Institute for Emergencies in Cardiovascular Diseases, Bucharest, Romania; “Carol Davila” University of Medicine and Pharmacy, Bucharest, Romania; *** Internal Medicine Department, Emergency Hospital of Bucharest, Bucharest, Romania; “Carol Davila” University of Medicine and Pharmacy, Bucharest, Romania; **** General Surgery Department, Emergency Hospital of Bucharest, Bucharest, Romania; “Carol Davila” University of Medicine and Pharmacy, Bucharest, Romania

**Keywords:** pulmonary arterial hypertension, congenital cardiac shunts, specific therapy

## Abstract

**Objective:** The aim of this study was to compare the effects of Sildenafil, Bosentan and combined therapy in patients with congenital cardiac shunts associated pulmonary artery hypertension (CCS-PAH).

**Design:** Prospective observational study (February 2011 – January 2014) with a historical control group (January 2009 – January 2011).

**Setting:** “CC Iliescu” Institute for Emergency Cardiovascular Diseases of Bucharest, a tertiary university-affiliated center.

**Patients:** All cases with CCS-PAH.

**Interventions:** Specific vasodilatory therapy: Sildenafil, Bosentan or combined therapy.

**Outcome Measures:** The primary outcome was the overall survival at 24 months.

**Results:** Out of 108 patients with pulmonary arterial hypertension, there were 79 patients with CCS-PAH, 55 presenting a severe form of the disease. The mean age of the patients was 34.42±21.15 years, with 37 (67,3%) female patients. 23 patients received specific vasodilatory treatment (thirteen Sildenafil, seven Bosentan, three combined treatment), with 32 patients in the control group, without specific vasodilatory therapy. The specific vasodilatory therapy was associated with improved WHO/ NYHA functional class (p=0.025), oxygen saturation at the end of the six-minute walk test (p=0.011), decreased pulmonary artery systolic (p=0.002) and diastolic (p=0.004) pressures, and an increased S’ wave in Tissue Doppler Imaging (p=0.008).

**Conclusions:** Despite the complexity of CCS-PAH, with a complex constellation of underlying congenital heart defects, there are short-term benefits of a specific vasodilatory therapy.

## Introduction

The prevalence of pulmonary arterial hypertension (PAH) in the general population is 5 to 15 cases per 1 million of adults [**1**]. Associated with congenital cardiac shunts (CCS), PAH is one of the most common causes of severe morbidity and premature mortality in patients with congenital heart diseases. Congenital heart diseases occur at a rate of 8 per 1,000 live births, as much as 30% of the patients with surgically unrepaired defects developing PAH [**2**]. In patients with surgically repaired congenital cardiac defects, PAH-CCS will appear in 15% of the cases [**3**]. The plasmatic levels of endothelin, a vasoconstrictor molecule with proliferation effects in the vascular muscular layer, are increased in patients with PAH-CCS [**4**]. The current evidence indicates the critical role of endothelin in the pathogenic mechanism of CCS-PAH, with a decrease in endothelin-1 after the surgical repair of the congenital cardiac malformation [**5**]. The biological and histological changes observed in these patients, such as lung vasculature endothelial dysfunction, are regarded as similar to those seen in idiopathic PAH and other forms of PH [**6**]. It should be noted that the patients with Eisenmenger syndrome present an increased survival and better hemodynamic parameters than those with idiopathic PAH [**7**]. The severe PAH includes patients with one of the following criteria: a) World Health Organization (WHO) functional class IV; b) inadequate response and/ or not meeting treatment goals; c) deterioration on maximal therapy [**8**].

## Methods

**The primary objective** of this study was to compare the effects of Sildenafil, Bosentan and combined therapy on the clinical status and echocardiographic parameters in patients with pulmonary hypertension associated with congenital cardiac shunts. 

**The secondary objectives were:** (a) To characterize the anatomical and pathophysiological changes of the right ventricle in CCS-PAH, (b) To establish the prognostic value of right ventricular dysfunction in CCS-PAH, by correlating it with survival free of adverse events at 24 months.

**Group of study:** We selected all the patients with PAH, admitted in “CC Iliescu” Institute for Emergency Cardiovascular Diseases of Bucharest, between January 2009 and January 2014. The study included a retrospective part, with patients admitted between January 2009 and January 2011 and a prospective observational component, with patients admitted between February 2011 and January 2014. All the patients from the control group were historical patients. 

**Inclusion criteria:** (1) Pulmonary arterial hypertension, defined as resting means pulmonary arterial pressure at or above 25 mmHg, pulmonary artery wedge pressure of less than 15 mmHg and pulmonary vascular resistance > 3 Wood units. These parameters were determined by right heart catheterization, regardless of the functional class and etiology. (2) PAH associated with congenital cardiac shunts. (3) Severe PAH: defined as pulmonary artery systolic pressure > 50 mmHg or likely PAH according to the European Society of Cardiology latest guideline [9].

**Exclusion criteria:** (1) Patients presenting with pulmonary hypertension other than those exposed in the inclusion criteria. (2) Patients with atrial fibrillation. (3) An improper echocardiographic window for evaluation.

**Follow-up of patients:** Patients were clinically and echocardiographically followed at a six-month interval, up to 24 months, trying to identify the adverse cardiovascular events: death, in-hospital admission for syncope or heart failure, worsening of the functional class with at least one WHO/ NYHA class. 

**Statistical analysis:** For the statistical analysis, patients were divided into four groups: (1) Sildenafil; (2) Bosentan; (3) Sildenafil and Bosentan; and (4) no treatment historical control group. Continuous variables were expressed as mean ± standard deviation, and the categorical ones as number (percent). After verifying the assumption of sphericity, with Mauchly’s test, Repeated Measures ANOVA was used for the analyses of clinical parameters and echocardiographic findings in patients under a specific vasodilatory therapy. Kaplan-Meier survival analysis was used. The probability of rejecting the null hypothesis (statistical significance) was set at 0.05. IBM SPSS Statistics 20 software was used for the statistical analysis. The patients’ verbal informed consent was formally obtained prior to each clinical evaluation.

## Results

A total of 108 patients with pulmonary arterial hypertension (PAH) were admitted during the study’s interval of five years. 79 patients had congenital cardiac shunts associated pulmonary arterial hypertension (CCS-PAH), with 55 patients presenting the severe form of the disease. The mean age of the patients was 34.42±21.15 years, 37 (67,3%) being female. Most of the congenital heart defects were found in a higher percentage of female patients, such as persistent arterial duct (PAD) in 87.5% of the cases, atrial septal defects (ASD) in 76% of the cases, anomalies of the pulmonary venous drainage in 70% of the cases. Ventricular septal defects (VSD) were found with an equal distribution between the two sexes (p = 0.024). The mean age of PAH patients with VSD was 23±13 years, 20 years lower than the ASD patients (44±23 years, p<0.05), and 15 years lower than anomalous pulmonary venous drainage patients (39±24 years, p<0.05). At the diagnosis of the congenital heart defect, patients with VSD had a mean age of 7.6 years, significantly lower than ASD (34 years) and PAD (14 years) patients (p<0.05). At the diagnosis of PAH in patients with VSD, the mean age was 12 years, significantly lower than that of patients with ASD (38 years), and with abnormal pulmonary venous drainage (28 years) (p<0.05). 

**Fig. 1:**
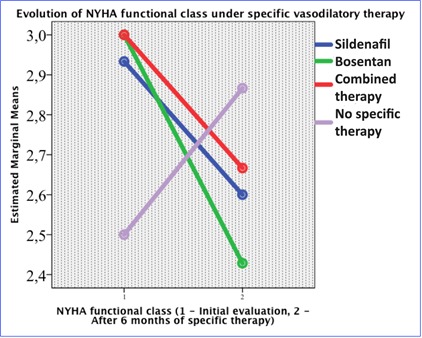
Evolution of NYHA functional class under specific therapy

Thirteen patients received Sildenafil as specific vasodilatory treatment, seven patients Bosentan and three patients associated treatment. 32 patients did not receive specific vasodilatory treatment and represent the historical control group. Nineteen patients had Eisenmenger syndrome, eight were included in the Sildenafil group, four in the Bosentan group, one in the associated treatment group and six in the no specific vasodilatory group. WHO/ NYHA functional class decreases with half class after six months of specific vasodilatory therapy in the Sildenafil, Bosentan or combination therapy groups, increasing in the no specific therapy group (p = 0.025). The patients in the Bosentan group had the most significant decrease of the WHO/ NYHA functional class (**Fig. 1**).

**Table 1:** Evolution of clinical parameters under specific vasodilatory treatment

	Received therapy	Initial evaluation	After more than 6 months	Statistical significance*
NYHA functional class	Sildenafil	2.9±0.3	2.6±0.5	*p = 0.025*
	Bosentan	3±0.6	2.43±0.5	
	Combined	3±0.1	2.67±0.6	
	No specific therapy	2.5±0.8	2.87±0.8	
Distance at six-minute walk test (meters)	Sildenafil	330.5±139.5	379.4±124.6	*p = 0.252*
	Bosentan	440±126.9	515±130.7	
	Combined	340.00±84.8	285±190.9	
	No specific therapy	396.7±55.1	420±72.1	
Oxygen Saturation at the beginning of the six-minute walk test (mm Hg)	Sildenafil	91.6±3.8	91.3±7	*P=0.018*
	Bosentan	84.8±7.5	89±6.8	
	Combined	78±19.8	90.5±4.9	
	No specific therapy	92.9±4.9	89.8±4.7	
Oxygen Saturation at the end of six-minute walk test (mm Hg)	Sildenafil	82.2±14.4	85.7±11.4	*p = 0.011*
	Bosentan	75.2±11.1	86±8.9	
	Combined	71±22.6	86.5±6.4	
	No specific therapy	80.7±19.2	80.3±19.1	
*The statistical test used was Repeated Measures ANOVA. The p-value reflects the statistical significance of baseline versus post-treatment comparison.				

**Fig. 2:**
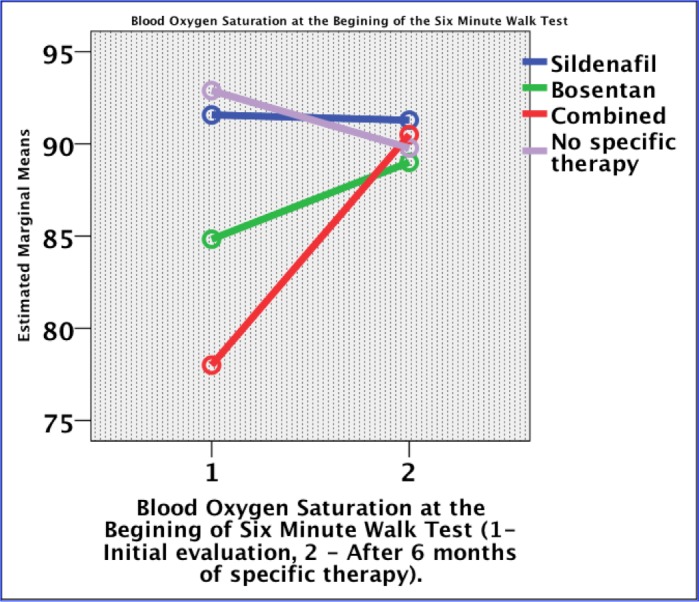
Evolution of oxygen saturation at the beginning of the 6-minute walk test under specific therapy

**Fig. 3:**
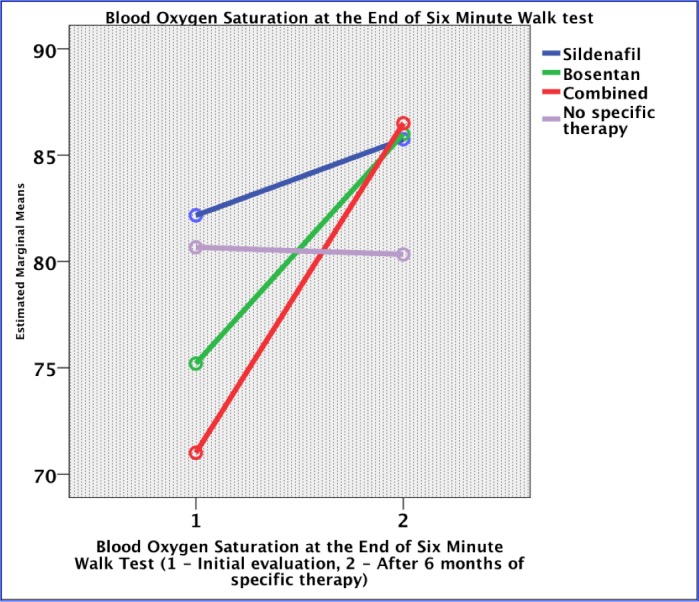
Evolution of oxygen saturation at the end of the 6-minute walk test under specific therapy

The distance of the 6-minute walk test (6MWT) increased after six months of Sildenafil or Bosentan but also in the control group (p= 0.252). The oxygen saturation at the beginning of 6MWT increased with 23 mmHg in the combination group and with 4 mmHg in the Bosentan group. There was no improvement in the Sildenafil group, while in patients without a specific treatment, the blood oxygen saturation decreased with a mean of two mmHg (p = 0.018) (**Fig. 2**). At the end of 6MWT, the oxygen saturation increased with 11 mmHg in Bosentan, 3 mmHg in Sildenafil and 15 mmHg in the combination therapy group. No improvement was noticed over time in the no specific therapy group (p = 0.011) (**Fig. 3**). 

The pulmonary artery mean pressure decreased with five mmHg in Sildenafil and two mmHg in Bosentan group (p = 0.597). 

**Table 2:** Evolution of echocardiographic parameters under specific vasodilatory treatment

	Received therapy	Initial evaluation	After more than 6 months	Statistical significance*
NYHA functional class	Sildenafil	56.7±9.9	51.5±11.3	*p = 0.597*
	Bosentan	61.7±9.2	59±9.2	
	No specific therapy	51.4±12.4	53.6±17.5	
Pulmonary Artery Systolic Pressure (mm Hg)	Sildenafil	96±18.7	89±17.1	*p = 0.002*
	Bosentan	108±23.5	88.7±24.6	
	Combined	90±8.5	70±14.1	
	No specific therapy	59±2.7	56.2±8.9	
Pulmonary Artery Diastolic Pressure (mm Hg)	Sildenafil	36.7±5.5	32.33±5	*p = 0.004*
	Bosentan	48.5±2.1	45±7.1	
	No specific therapy	31.2±14.4	31.7±14.9	
TAPSE (mm)	Sildenafil	17.6±4.3	19±4.2	*p = 0.583*
	Bosentan	20.2±4.6	21.7±4.2	
	Combined	17.3±6.6	20±3.5	
	No specific therapy	21.7±6.1	18.3±3.2	
Right atrium area (cm2)	Sildenafil	12.2±4.8	13.2±6.5	*p = 0.715*
	Bosentan	14.9±2.6	14±1.5	
	Combined	9.5±4.9	10.7±6.1	
	No specific therapy	29±11.5	25.3±7.1	
S’ wave in the Tissue Doppler Imaging (cm/ s)	Sildenafil	10.2±1.2	11.2±1.1	*p = 0.008*
	Bosentan	10	11.5	
	No specific therapy	17	7	
*on Repeated Measures ANOVA, as the statistical test used. The p-value reflects the statistical significance of baseline versus post-treatment comparison.				

**Fig. 4:**
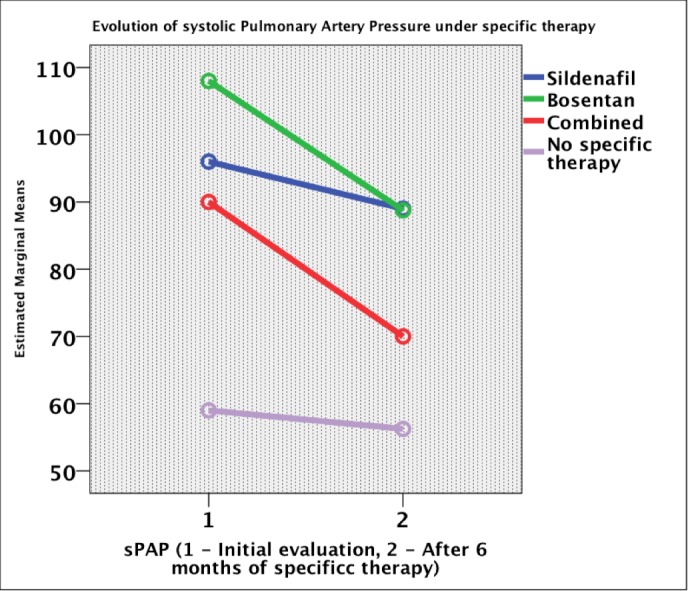
Evolution of pulmonary artery systolic pressure under specific therapy

**Fig. 5:**
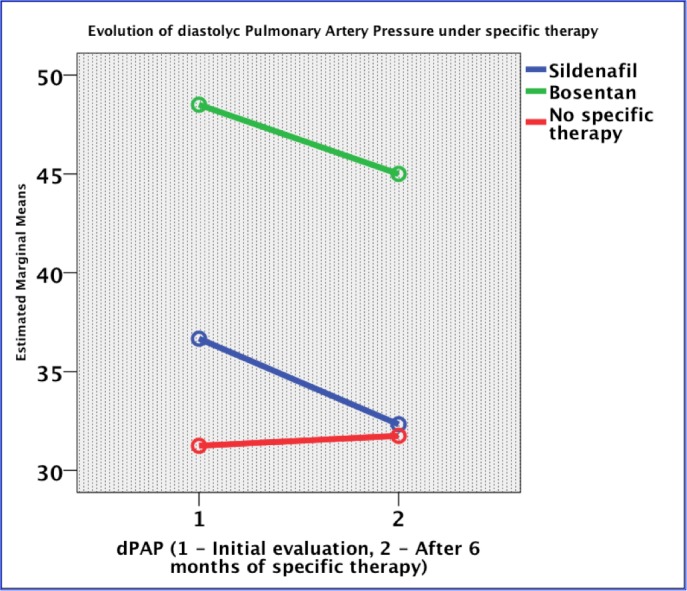
Evolution of pulmonary artery diastolic pressure under specific therapy

The pulmonary artery systolic pressure decreased with five mmHg in the Sildenafil group, 20 mmHg in Bosentan and combination therapy groups, and three mmHg in patients with no specific therapy (p = 0.002). After 24 months of combination therapy, the pulmonary artery systolic pressure decreased with 22 mmHg compared to the initial value and increased with four mmHg in no treatment group (p = 0.012) (**Fig. 4**). The pulmonary artery diastolic pressure decreased with four mmHg in Sildenafil and Bosentan groups, with no modifications in no specific therapy group (p = 0.004) (**Fig. 5**). The pulmonary acceleration time increased with 22 ms in Bosentan, 4 ms in the Sildenafil group and 30 ms in no specific therapy group (p = 0.224). TAPSE increased with 1 mm in Bosentan and Sildenafil groups, with 3 mm in the combination therapy, and decreased with 3 mm in no specific therapy group (p = 0.583). The tricuspid regurgitation decreased with half a class in the Bosentan group and did not change in the Sildenafil and combination therapy groups. The S’ wave at the level of the tricuspid annulus increased with 1 cm/ s in the Sildenafil and 2 cm/ s in the Bosentan groups and decreased with 5 cm/ s in no specific treatment group (p = 0.008). The E’ wave analysis at the level of the tricuspid annulus increased with 3 cm/ s in specific therapy groups (p = ns). The A’ wave at the level of the tricuspid annulus increased with 2 cm/ s in the Sildenafil and Bosentan groups (p = ns). The right ventricular end-diastolic diameter decreased in all groups of specific vasodilatory therapy and increased with four mm in patients without specific treatment (p = 0.644). 

There was a mortality rate of 5.5% during the study interval. The Kaplan-Meyer analysis showed no statistically significant difference between the study groups (p = 0.079). 

**Fig. 6:**
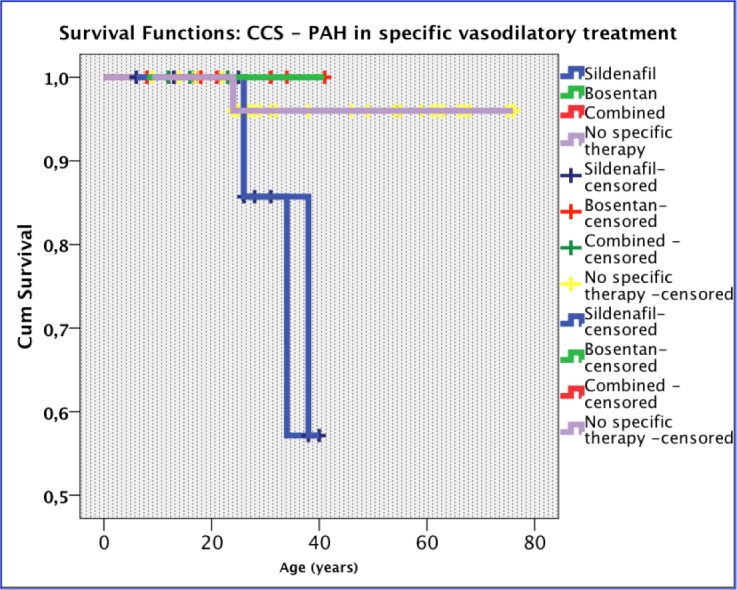
Kaplan-Meyer survival analysis

## Discussions

 Our study revealed that specific vasodilatory therapy was associated with a significant improvement of clinical and echocardiographic parameters in patients with severe pulmonary hypertension due to congenital heart diseases. Echocardiography was particularly suited for the dynamic evaluation of these patients. According to the current evidence, a worse prognosis was suggested by the presence of clinical data of right ventricle failure, rapid progression of symptoms, syncope, and WHO functional class IV. Additional negative prognostic factors are a distance shorter than 300 m on 6MWT, a peak Oxygen consumption < 12 mL/min/kg on cardiopulmonary exercise testing, elevated and increasing BNP/ NT-proBNP plasma levels, pericardial effusions and TAPSE < 1.5 cm on echocardiography and right atrial pressure > 15 mmHg or cardiac index ≤ 2 L/min/m2 on cardiac catheterization [**10**]. Combination therapies, used to maximize the efficacy and minimize toxicity, seem to be associated with better outcomes when predefined treatment goals are followed [**11**]. Only a careful orchestrated goal-oriented therapy may offer a satisfactory clinical status [**12**].

***The prostaglandin analogs.*** Epoprostenol improves the hemodynamic parameters and functional capacity when administered in patients with PAH and congenital heart disease. The current evidence supports the use of epoprostenol as first-line therapy in patients with severe disease, WHO/ NYHA class IV, given that only a few patients with severe disease were included in studies with alternative agents [**13**]. Treprostinil has some administration advantages compared with epoprostenol, since it may be given subcutaneous continuously, has a greater half-life and requires no refrigeration [**14**]. Olschewski et al. reviewed the results of the treatment with inhaled iloprost in patients with severe PAH, WHO/ NYHA class III or IV [**15**]. There was an increase in the distance to the 6-minute walk test of 36.4 m, with significant improvements in the WHO/ NYHA class, dyspnea, and quality of life [**15**].

***Endothelin receptor antagonists.*** Bosentan, a non-selective endothelin receptor antagonist with oral administration, improves hemodynamic parameters, exercises capacity, and reduces the clinical deterioration [**16**]. Baptista et al. followed 14 patients with PAH and congenital cardiac diseases for a median duration of 4 years [**17**]. Patients had a mean age of 37.1±11.7 years, 90% in the WHO/ NYHA class III and IV. Congenital heart diseases were the pulmonary atresia associated with ventricular septal defect (35.7%), common arterial trunk (28.6%), persistent arterial duct (21.4%) and transposition of great vessels (14.3%). After six months of treatment, the distance from the 6-minute walk test increased from 371.9 to 428.4 m (p = 0.005), with the improvement of the functional class. After four years, the average distance at the 6-minute walk test in patients treated with Bosentan or Bosentan and Sildenafil was 440.1±103.8 m, and 428.8±96.9 m, respectively. The authors concluded that, the favorable effects of Bosentan therapy persist up to 4 years in complex congenital heart diseases [**17**]. 

The mean age of our patients was 34.42±21.15 years. Out of 55 patients with CCS-PAH in our study, thirteen cases received Sildenafil, seven received Bosentan and three combined therapy. After 6 months of specific vasodilatory therapy, we have observed a half class decline for WHO/ NYHA functional class, a 100 m increasing the distance at the 6-minute walk test, and a marked increase for the Oxygen saturation at the beginning and at the end of the 6-minute walk test. 

Ambrisentan is a selective antagonist of the endothelin receptor type A, which can also be administered orally. Galie et al. published the results of ARIES 1 and 2 study, a randomized multicenter study regarding the Ambrisentan therapy [**18**]. The authors concluded that therapy with Ambrisentan improved exercise capacity and is well tolerated, with a low risk of increasing in liver transaminases [**18**]. The same group published the effects of long-term therapy with Ambrisentan [**19**]. After 2 years of treatment, the distance from the 6-minute walk test improved by 23 meters (5 mg dose) and 28 meters (10 mg). The survival rates and the absence of clinical deterioration at 1 year was 94% and 83% respectively, and, at 2 years, these parameters were 88% and 72% [**19**]. 

***Phosphodiesterase type 5 inhibitors.*** Galie et al. evaluated the treatment with Sildenafil in 278 patients with symptomatic PAH [**20**]. Patients treated with Sildenafil had a distance at the 6-minute walk test greater with 45 m (20 mg Sildenafil, 3 times per day), 46 m (40 mg Sildenafil, 3 times per day) and 50 m (80 mg Sildenafil, 3 times per day). The patients from all the three treatment groups (20, 40, 80 mg) showed improvements in the pulmonary artery mean pressure and WHO/ NYHA functional class [**20**]. We also observed improvements of the mPAP and WHO/ NYHA functional class for patients receiving Sildenafil. 

***Combination therapies.*** Benza et al. evaluated the long-term efficacy of subcutaneous Treprostinil therapy, and its combination with Bosentan, in patients with moderate and severe PAH [**21**]. The analysis of 38 patients showed that the mean pulmonary arterial pressure decreased from 59.7 to 50.5 mmHg, with a significant improvement in the distance to 6-minute walk test and Borg dyspnea score. If initially, less than 2% of the patients were in the functional class II or less, at the latest clinical evaluation, 58% of the patients were in this class. The addition of oral Bosentan therapy in 19 patients came with further improvements in the hemodynamic and functional parameters [**21**]. In our study, only 3 patients received combined therapy of Bosentan and Sildenafil. Moreover, the mean PAP decreased with 5 mm for the Sildenafil group, 2 mm for the Bosentan group, while for patients without a specific therapy, a 2 mm increase was observed.

In the CCS-PAH patients, the only drug evaluated in a prospective randomized study, compared to the placebo treatment, is Bosentan [**22**]. The conclusion of this study is that Bosentan was well tolerated and improves exercise capacity and hemodynamic parameters without compromising peripheral oxygen saturation [**23**]. D’Alto et al. evaluated the benefits of therapy with Bosentan and Sildenafil in patients with Eisenmenger syndrome and concluded that the combination therapy improves the clinical status, peripheral oxygen saturation at effort and hemodynamic parameters [**24**]. A systematic review published in Cochrane database concluded that the endothelin receptor antagonists might increase exercise capacity, improve the WHO/ NYHA functional class, prevent the deterioration of WHO/ NYHA functional class, reduce dyspnea and improve the hemodynamic parameters. However, their impact on reducing mortality is low and the level of evidence of the studies is stronger for the idiopathic PAH [**25**]. On the other hand, the right ventricular dysfunction represents the leading cause of death in patients with pulmonary hypertension. However, there is insufficient data regarding the effect of PAH treatment on the right ventricular geometry and function, knowing its particular spatial geometry, difficult to assess by usual imagistics [**26**]. The echocardiographic parameters, such as, right ventricular fractional area change, and the right ventricular global strain value are correlated with the prognosis of patients with PAH under specific therapy [**27**]. Our study showed a 20 mmHg decrease of the sPAP in Bosentan and combined therapy groups (p = 0.002), with a four mmHg lowering of the dPAP in patients receiving Sildenafil and Bosentan (p = 0.014). The S’ wave at the level of the tricuspid annulus increased with one cm/s in Sildenafil and with two cm/ s for the Bosentan group (p = 0.004).

The pathophysiological mechanism of PAH is different between ASD and VSD patients. The pulmonary circulation is affected by the magnitude and duration of the volume overload in the ASD patients, while in patients with VSD, the pressure is added to the volume overload. In patients with ASD, the cardiac shunt occurs after the maturation of the pulmonary vessels, so they may compensate the volume overload by vasodilatation and recruitment of the new vessels. In this case, the pulmonary arterial pressure increases markedly in adulthood. In contrast, patients with a large (nonrestrictive) VSD have severe PAH at birth, the pulmonary vascular resistance becoming stable in childhood, often leading to the reversal of the shunt and Eisenmenger syndrome [**28**]. In our study, we found a mean age for the diagnosis of CCS-PAH in patients with VSD of 12 years, significantly lower than that of patients with ASD (38 years), and with abnormal pulmonary venous drainage (28 years) (p<0.05).

A review of the literature investigating the effects of endothelin antagonists, published by Liu et al. in 2013, includes 12 randomized trials and 1471 patients[**29**]. All included studies have a duration between 12 weeks and 6 months. After the treatment, patients treated with the endothelin receptor antagonists had a 33.71 m longer distance at the 6-minute walk test than patients treated with placebo, an improved WHO/ NYHA functional class (odds ratio = 1.6) and a decreased rate of clinical deterioration (odds ratio = 0.26). There was a reduction in mortality, which did not reach statistical significance. Regarding the Sitaxsentan, some cases of irreversible liver failure were noticed, with its withdrawing from the clinical use [**29**]. Rosenzweig et al. evaluated the results of Prostacyclin (PGI2) therapy in 20 patients with CCS-PAH, with a mean age of 15±14 years [**30**]. None of the patients had an acute response to therapy with PGI2, the pulmonary artery mean pressure decreasing with 21% to chronic administration (from 77±20 to 61±15 mmHg, p < 0.01). The pulmonary index improved from 3.5±2.0 to 5.9±2.7 L/min/m2 (p < 0.01) and the pulmonary vascular resistance from 25±13 to 12±7 U/ m2 (p < 0.01). The WHO/ NYHA functional class improved from 3.2±0.7 to 2.0±0.9 (p < 0.0001). The exercise capacity increased from 408±149 to 460±99 m (p = 0.13). The authors concluded that the chronic administration of PGI2 improves hemodynamic parameters and the quality of life in patients with CCS-PAH [30].

***Study limitations***

A limitation of this study might be the small number of patients in each subgroup of congenital diseases that are at the origin of PAH, with a relative etiological heterogeneity. The non-randomized nature of the study, together with the small groups size, might explain the lack of statistical significance regarding the survival data. What should be emphasized is that most of the literature is based on studies that also include a small number of patients, frequently below 30. Another limitation is the bidirectional type of the study, with a prospective and retrospective inclusion of the cases and the lack of randomization, which cannot be applied. The control group of untreated patients was investigated only before the starting of the national program of specific vasodilatory therapy in PAH, and there is a high risk of selection bias. The follow-up protocol did not include data derived from cardiac catheterization. However, we evaluated the pulmonary artery systolic pressure derived by echocardiography at each follow-up clinical evaluation.

## Conclusions 

Despite the results, CCS-PAH represents a complex condition, associated with a very wide and heterogeneous constellation of underlying congenital heart defects, the short-term benefits of specific vasodilatory therapy being quite clear. For patients with severe CCS-PAH, the specific vasodilatory treatment produced a significant improvement of the clinical status (WHO/ NYHA functional class, walked distance at the 6-minute walk test, oxygen saturation) and of the echocardiographic parameters (decrease of the pulmonary arterial systolic pressure, increasing of the S’ wave at the level of the tricuspid annulus, right ventricular end-diastolic volume). Dedicated registries for these patients include the required number of patients in order to reach the statistical significance regarding the impact of a specific therapy on the patients’ mortality. 

**Acknowledgment**

No external financial support. 

**Declaration of Interest**

The authors report no conflicts of interest. 
